# Early upregulation of immune suppressors dominates the macrophage response to *Toxoplasma gondii*

**DOI:** 10.1371/journal.pone.0336849

**Published:** 2025-11-24

**Authors:** Dominykas Murza, Filip Lastovka, George Wood, Matthew P. Brember, Oliver Chan, James W. Ajioka, Betty Y. W. Chung

**Affiliations:** 1 Department of Pathology, University of Cambridge, Cambridge, United Kingdom; 2 Institute of Biotechnology, Life Sciences Center, Vilnius University, Vilnius, Lithuania; Freie Universität Berlin, GERMANY

## Abstract

The apicomplexan parasite *Toxoplasma gondii* is a master manipulator, subverting its host through secreted proteins, hormone disruption, and even behavioural changes. Macrophages, the immune system’s first responders, play a pivotal role in determining infection outcomes, yet the initial triggers shaping these complex responses remain elusive. This study unveils the earliest transcriptional shifts in a mouse macrophage-like cell line RAW264.7-*T. gondii* infection model. Using time-resolved transcriptomic profiling, we captured host and parasite gene expression dynamics within the critical 15–120 minute window — when the host mounts its first line of defence and the parasite secures its foothold. By leveraging inactivated parasites, we disentangled responses to active invasion from general immune activation. By 60 minutes, macrophages exhibited a trend of increased suppressor of cytokine signalling expression — uniquely tied to live infection — while stress and pro-growth genes became dysregulated. Meanwhile, *T. gondii* responded with a slow but strategic transcriptional shift: an early increase in transcription and growth capacity, followed by a delayed activation of secreted proteins. These findings reveal a tug-of-war at the transcriptional level, where macrophages show rapid upregulation, while *T. gondii* employs a measured, delayed strategy to carve out its replicative niche.

## Introduction

*Toxoplasma gondii* (hereafter referred to as *T. gondii* or *Toxoplasma*) is a protozoan intracellular parasite that infects warm-blooded animals. Its importance is illustrated by its very high prevalence, with estimated 25.7% of global human population being seropositive [1]. *T. gondii* is able to infect immune-privileged organs, such as eye or brain [2,3]. Between the first exposure and the resulting tissue damage, many layers of regulation of cellular function, studied to different extent, act simultaneously, adding up to difficult-to-predict infection outcomes. We hypothesize that transcriptome disturbance during the infection event and immediately afterwards plays a substantial role in determining its outcome.

Previous studies have addressed multiple aspects of transcriptomics after infection by *T. gondii.* While *Toxoplasma* is known to infect a diverse repertoire of cell types, there are indications that transcriptional response to infection depends on the host cell type [4]. For example, in Vero cells, several infection-induced apoptosis pathways were activated [5]. Infection of human foreskin fibroblasts (HFF) by *T. gondii* bradyzoites resulted in upregulation of c-Myc, NF-κB and energy-related pathways [6]. *In vivo,* transcriptomic analyses of *T. gondii-*infected mouse brain tissue showed that infection disrupts normal expression patterns of genes associated with neurological functions [7], and that this observation also held true *in vitro* [8]. Another *in vivo* mouse brain study also demonstrated immune-related gene upregulation at both 11 and 33 days post infection with *T. gondii* oocysts [9]. Similar studies examined a variety of other organs, including testes and uterus [10], lung, lymph nodes and spleen [11], brain [12], and liver [13].

However, abundant lines of evidence point at the importance of different cell types, especially macrophages during infection by *T. gondii* [14]. The lineup of microneme (MIC), dense granule, and rhoptry proteins (ROP) secreted into the host upon infection has received particular attention. For example, the protein MIC3 caused increased TNF-α production and M1 macrophage activation [15]. The dense granule protein TgIST reaches the host cell nucleus to repress STAT1-dependent promoters [16,17]. ROP5, ROP17 and ROP18 collectively inhibit Immunity-Related GTPase (IRG) recruitment to the parasitophorous vacuole (PV) [18]. ROP16 can induce activation of STAT3 and STAT6, thereby initiating M2 polarization, a program with reduced anti-microbial capacity, to which suppressed generation of reactive oxygen species (ROS) is known to contribute [19]. Of note, macrophages can be subdued even without being infected, as secretory protein injection can occur regardless [20]. Finally, macrophages and their precursors, monocytes, also function as vehicles for facilitated migration of the parasite within the host’s body [21,22]. The modulation of migratory potential further highlights the importance of studying macrophage gene expression changes during *T. gondii* infection. Previous transcriptomic evidence of immune cell interactions with *T. gondii* by Lee et al. showed that pre-infecting macrophages before LPS treatment broadly dampens the immune response [23]. Additionally, it was demonstrated that type I strain RH perturbed the transcription of mouse bone marrow-derived macrophages (BMDMs) more markedly than the type II strain PTG, including stronger induction of macrophage migration-associated genes [24]. Yet, early macrophage transcriptomics upon *T. gondii* infection has not been previously covered in greater detail.

In the intricate interplay between host and pathogen during entry, understanding the temporal dynamics of gene expression is crucial. Distinct alterations to both host and *Toxoplasma* transcriptomes were observed in various time scales following infection. In particular, expression patterns were previously analyzed from two hours to 32 days post-infection and beyond [4–8,11–13,24–26]. Notably, most studies to date have concentrated on the pre-infection conditions and the experimental endpoints, leaving the dynamic events occurring in between largely unexplored. Significant changes in the host transcriptome are anticipated within the first two hours post-infection, as demonstrated by studies involving viral [27], bacterial [28], and eukaryotic pathogens [29]. However, no comparable early-infection transcriptomic time-course data have been published for *T. gondii* or any host cell type it infects. We expect that during this critical period of initial interaction, the transcriptomes of *Toxoplasma* and macrophage undergo primary changes that propagate the response via effector and regulator molecules and ultimately contribute to determining the parasite’s establishment within the host. However, studying related phenomena *in vivo* is not feasible due to sparse infection and imprecise timing control. Our *in vitro* study design addresses asynchronous infection and dilution of signal caused by low infection rates. The obtained results highlight the importance of global transcriptional remodeling by *T. gondii* infection, particularly on early onset of signaling and transcription factors. We speculate that the observed extensive immediate transcriptional response directs further course of macrophage infection by *T. gondii.*

## Results

### Establishment of efficient *in vitro* infection

A caveat of many past experimental designs is the unknown degree of confounding influence on the transcriptome caused by non-specific activation (e.g., due to temperature changes or extracellular factors). It is known that macrophage transcriptome undergoes drastic changes in early-response cytokine gene expression as soon as one hour post-lipopolysaccharide (LPS) stimulation [30]. Likewise, *in vitro* infections are prone to include the non-specific stimulation component caused by lysed *T. gondii* in the medium. Extracellular single-stranded RNA, for instance, is expected to be present in such lysates and is known to activate Toll-like receptors (TLR) 7 and 8 in mice and humans, respectively [31]. Additionally, in the human myelomonocytic cell line THP-1, *T. gondii* lysate was also able to trigger immunosuppression via TLR2-mediated NF-κB activation [32]. Our design implements fully inactivated *T. gondii* lysate controls (further referred to as Dead *Toxoplasma*; Fig S1 in S1 File) to estimate the extent of and adjust for the side effects of non-specific stimulation during *in vitro* infections. It is important to note that the Dead *Toxoplasma* condition uses a lysed parasite suspension, therefore, unlike in the Live *Toxoplasma* infection condition, the host cells are exposed to both internal and external components of the parasite. Also, media-only controls (Mock) are used to account for any possible signal due to temperature fluctuations, mechanical stress etc.

**Fig 1:**
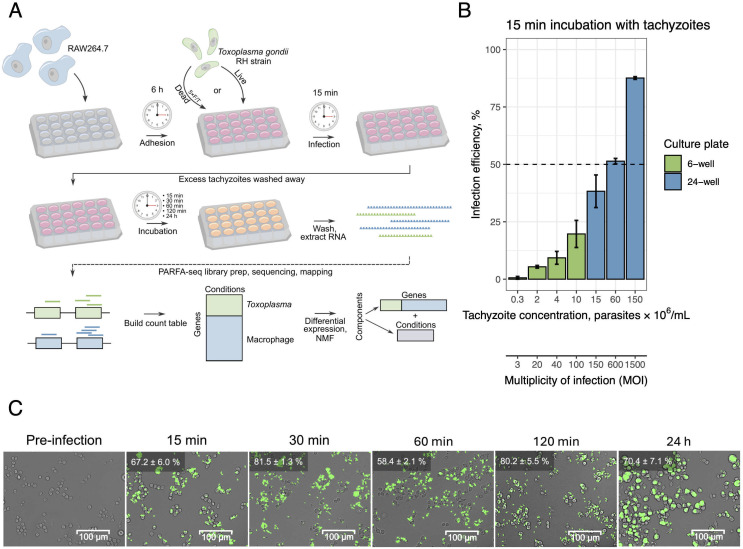
A. Experimental scheme of infection time course and downstream workflow; B. Optimization of tachyzoite concentration to achieve adequate infection rates, with a threshold set at 50% infection efficiency. Error bars represent ± 1 standard deviation. Corresponding MOI values are given for each condition; C. Infection time course micrograph merged from brightfield and green channels. *T. gondii* exhibits green fluorescence due to stable GFP expression in the strain used. Percentage at the top left indicates infection efficiency (± 1 standard deviation) for each time point.

The experimental workflow is outlined in Fig 1A. For the procedure, we selected a common model mouse macrophage cell line RAW264.7 to facilitate comparison to previous studies [15,33,34] as well as Type I GFP-tagged *T. gondii* strain RH. We aimed to achieve adequate infection synchronicity as a prerequisite for this workflow. Therefore, the duration of parasite suspension exposure to RAW264.7 was set to 15 min to match the time scale of intended sampling resolution. Due to this limiting timeframe of infection, we chose to express parasite quantity in terms of concentration. In this design, the commonly adopted multiplicity-of-infection (MOI) metric may be overestimating as most parasites do not sediment through the media column quick enough to get in contact host cells. Parasite concentration in a fixed volume of medium during infection was then optimized to yield at least 50% infection efficiency (Fig 1B). Then, the selected concentration of 6 × 10^7^ tachyzoites/mL was used for the infection time course (15, 30, 60, 120 min and 24 h). For each time point, RAW264.7 cells were treated with either *Toxoplasma*-free medium (Mock), live *T. gondii* (Live *Toxoplasma*) and parallel dead *T. gondii* through successive freeze thaw cycles (Dead *Toxoplasma*) in triplicate. For the Live *Toxoplasma* condition, threshold infection efficiency was achieved at every time point, with occasional host cells being infected by more than one parasite cell (Fig 1C). At 24 hours, increased burden of parasites leading to drastic changes in RAW264.7 morphology was observed. In parallel to imaging, sample total RNA was harvested, its quality was confirmed (Fig S2 in S1 File), and two replicates were used to prepare PARFA-seq libraries.

### Polyadenylated RNA Fragment Abundance Sequencing library preparation and composition analysis

**Fig 2:**
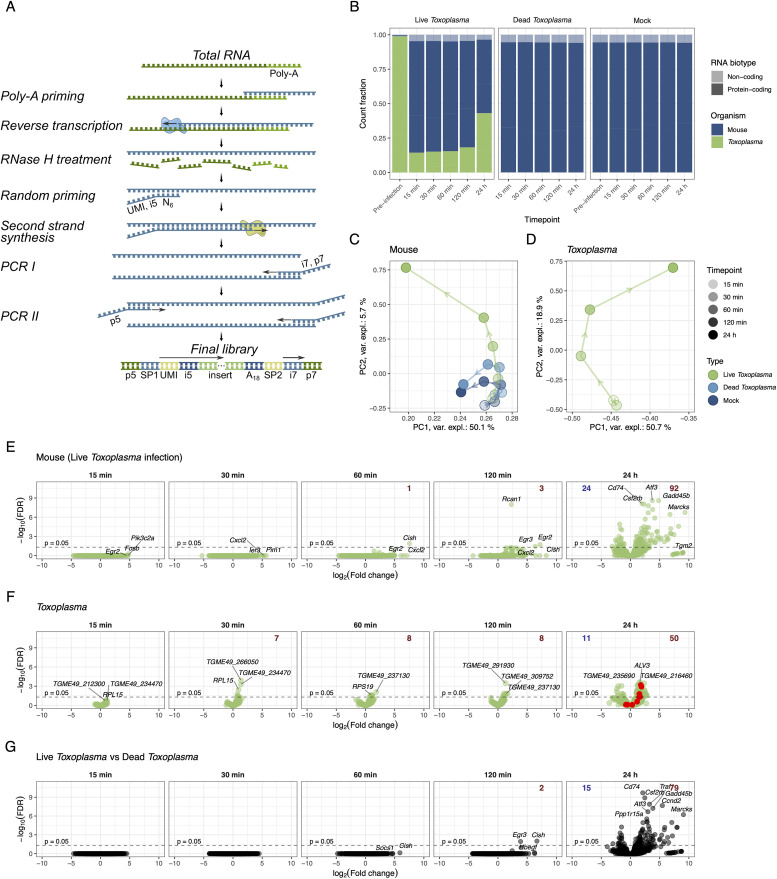
A. Library preparation strategy: poly-A(+) RNAs are captured by complementarity to reverse transcription primer, then second strand is synthesized using random hexamers and handles for sequencing are attached through two rounds of PCR. B. Mapped read distribution by species and by biotype. C and D. Principal component analysis of mouse and *T. gondii* transcriptomes, respectively. E and F. Volcano plots of Live *Toxoplasma*-infected mouse and *T. gondii* time points, respectively. To calculate Log_2_(Fold change), every timepoint is compared to corresponding samples (either macrophage or *Toxoplasma*) before infection (t = 0 min). Most significant and most upregulated transcripts are labelled. Number of significantly (p ≤ 0.05) downregulated and upregulated genes for each time point is given in blue and red, respectively. In F, 24-hour time point, red dots mark ROP genes. G. Volcano plot for macrophage transcriptomes when corresponding timepoints of Live *Toxoplasma* and Dead *Toxoplasma* are compared.

PolyAdenylated RNA Fragment Abundance (PARFA)-Seq was established to facilitate our understanding of transcriptome dynamics during *T. gondii* infection of macrophages. PARFA-seq library preparation workflow is illustrated in Fig 2A.

Mapped read classification by species and RNA biotype (Fig 2B) demonstrates species- and biotype-specific alignment. Additionally, increasing fraction of *T. gondii* transcript counts throughout the time course confirmed proliferation of the parasite and agrees with the micrographs (Fig 1C) and the total RNA profiles (Fig S2 in S1 File). Also, macrophages incubated with Dead *Toxoplasma* did not contain *T. gondii* RNA, confirming successful inactivation of *T. gondii*. Global transcription assessment through principal component analysis (PCA) revealed Live *Toxoplasma*-infected macrophage transcriptome notably diverged from both control conditions by 60 min (Fig 2C). Although a high MOI was used, no strong transcriptional response was evident prior to this timepoint. In parallel, significant change of *T. gondii* transcriptome at 60 min was also observed, whereas global difference between 15 min and 30 min was less pronounced (Fig 2D). Differential expression analysis was conducted by comparing each time point to the pre-infection state (t = 0; Fig 2E and F, S5 Fig in S1 File). Notably, transcriptional upregulation was more prominent than RNA decay for both the host and the parasite throughout the course of infection, as shown by higher numbers of significantly upregulated genes. In macrophages, typical early response genes that modulate inflammation (*Egr2, Fosb, Cxcl2, Cish*) had high Log_2_(Fold change) values. In addition, comparing macrophage transcriptomes directly between Live and Dead *Toxoplasma* conditions for every timepoint enabled precise adjustment for non-specific stimulation, revealing that the suppressor of cytokine signaling response (*Cish*, *Socs1*) is specific to Live *Toxoplasma* infection (Fig 2G). Among the highly upregulated *T. gondii* genes, no clear patterns or commonalities emerged throughout the time course. Statistically significant changes in *Toxoplasma* transcriptome were modest between 15–60 min post infection indicating similarity to the control condition – pre-infection *Toxoplasma* (Fig 2F).

### *Toxoplasma* transcriptional landscape during macrophage infection

**Fig 3:**
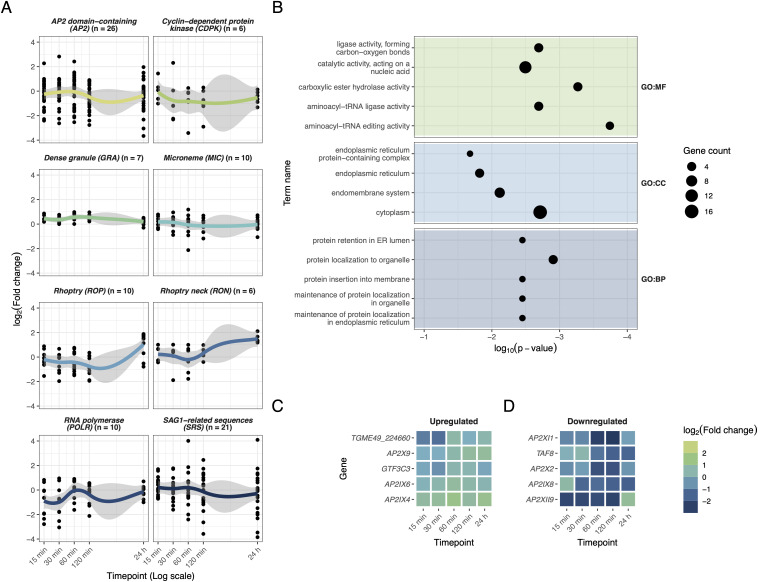
A. *T. gondii* gene group changes throughout the time course, expressed as log_2_(fold change) relative to pre-infection tachyzoites (t = 0). Grey area depicts the 95% confidence interval. n value refers to number of genes detected in each group. B. Gene set enrichment plot for most enriched terms from *T. gondii* transcriptome features at 15 min to 120 min. Five most significant terms are displayed for each database. C and D. Transcriptional profiles of top five upregulated and downregulated *T. gondii* transcription factors, respectively.

The behavior of eight groups of *T. gondii* genes known to be important for infection and parasite proliferation was initially examined throughout the time course. Fold changes at each time point were calculated relative to the pre-infection condition (time zero) to account for baseline expression and allow direct comparisons across all conditions and time points (Fig 3A). Macrophage RNA for the pre-infection condition was harvested right before addition of *T. gondii* suspension while for *T. gondii* itself, RNA was extracted from the suspension of tachyzoites used to infect. Among the most populous gene groups shown, *T. gondii* underwent an increase in RNA polymerase gene expression at 60 min. Rhoptry and rhoptry neck (RON) protein transcripts were upregulated at 24 hours (Fig 2F, Fig 3A).

An independent Non-negative Matrix Factorization (NMF) analysis was additionally performed on macrophage-*T. gondii* metatranscriptome from 15 to 120 minutes post-infection (excluding the 24-hour timepoint) to disentangle early responses (Fig S6 in S1 File). Genes associated with transfer RNA charging function, cytoplasm localization and membrane-associated proteins were upregulated at early time points (Fig 3B). Additionally, the differential expression of *T. gondii* transcription factors appeared relatively modest, with log_2_(fold change) of all genes falling within −3 to 3 (Fig 3C and D). Collectively, these findings suggest an increased capacity for growth and reproduction within the host, as well as preparation for subsequent infection.

### Macrophage transcriptome response

**Fig 4:**
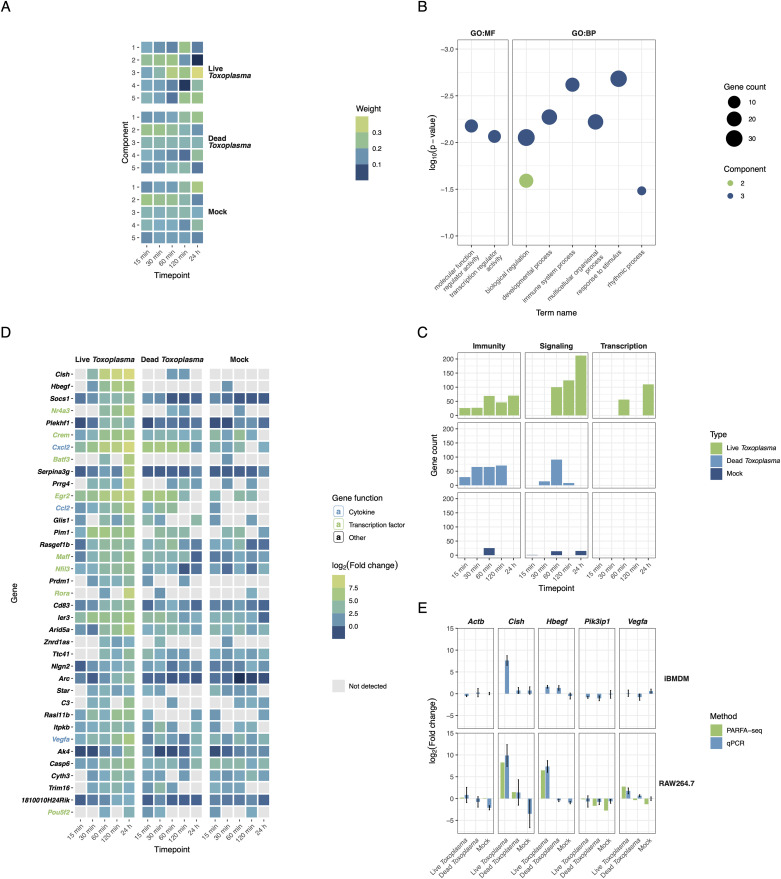
A. Mouse transcriptome non-negative matrix factorization component weights across all time points and conditions. Larger weight values indicate components with upregulated genes dominating. B. Gene set enrichment plot for selected features from each component in panel A. Components 1, 4, and 5 did not exhibit enrichment in these broad terms. C. Number of genes in enriched gene ontology terms related to immunity, signaling, and transcription for each time point and condition. D. Time course transcriptional profiles for mouse genes characteristic to component 3. E. PARFA-seq validation by quantitative PCR and gene expression changes during immortalized bone marrow-derived macrophage infection by *T. gondii* assessed by quantitative PCR.

We next focused on the macrophage response. In agreement with principal component analysis, NMF analysis showed subtle differences between Dead *Toxoplasma*-stimulated macrophages and mock-treated cells, whereas Live *Toxoplasma* condition was clearly distinct in component 3 (Fig 4A). Gene set enrichment analysis of the driving features in each component (Fig S7 in S1 File, Fig 4B) revealed that functional enrichment – including responses to stimuli, transcription regulation, and immune processes – is concentrated in component 3.

To investigate the dynamics of immunity-, signaling-, and transcription-related genes, we focused on genes with a log_2_(fold change) above two in each condition and counted those belonging to the enriched terms (Fig 4C and S2 Table). Transcription- and signaling-related gene enrichment was most pronounced in Live *Toxoplasma*-treated macrophages. However, during first two hours, Dead *Toxoplasma* also induced a comparable range of immune genes, in some cases preceding the response in Live *Toxoplasma-*infected macrophages.

Features characteristic of component 3 include suppressors of cytokine signaling, *Cish* and *Socs1*, both of which underwent marked upregulation, specific to Live *Toxoplasma* infection (relative to Dead *Toxoplasma* treatment) by 30 minutes (Fig 4D). This observation corroborates previous evidence that *Cish* and *Socs1* are upregulated at four hours post-infection [35]. Interestingly, both Live and Dead *T. gondii* treatments trigger chemokine (*Cxcl2*, *Ccl2*) upregulation, although peak levels of these transcripts were detected later in case of live *Toxoplasma* infection. Among the main transcription factors driving the early (<60 min) transcriptional response is *Nr4a3*, in line with previous findings [36].

Finally, expression pattern of a range of genes, including *Cish,* identified through PARFA-seq were validated by qPCR, underscoring the robustness of PARFA-seq in detecting transcriptional changes (Fig 4E). To further support our findings on macrophage response, we evaluated the same targets by qPCR in a parallel infection time course using murine immortalized bone marrow-derived macrophages (iBMDMs). Among the targets tested, *Cish* expression most closely mirrored the macrophage response to *T. gondii* infection, while other transcripts showed only partial or inconsistent trends.

## Discussion

In this work, we developed and applied PARFA-seq to simultaneously investigate the transcriptomic responses of both mouse macrophages and *T. gondii* immediately following invasion. Our results show that macrophages undergo a pronounced transcriptional response within the first 30–60 minutes of parasite contact (based on Log_2_(Fold change)). These early changes are likely to be crucial in shaping the subsequent course of infection by suppressing host innate immunity to aid in establishment within the macrophage.

We have identified a set of genes involved in immune response, signaling and transcription regulation that act early in the infection timeframe. In line with previous findings [35], suppressors of cytokine signaling – *Cish* and *Socs1 –* were upregulated. In particular, this upregulation was dependent on successful internalization and further proliferation of the parasite; the factors released during lysis (Dead *Toxoplasma* condition) were not sufficient to produce an equivalent response. The well-established function of these gene products is to provide negative feedback and prevent overactivation of the immune system [37]. However, they are also apparent targets for exploitation by various pathogens including Avian leucosis virus [38], Human immunodeficiency virus [39] or *Listeria monocytogenes* [40]. In under 30 min post-infection by the *T. gondii* RH strain, expression of another gene of the group, *Socs3,* was also observed at the protein level [41], likely reflecting manipulation by the parasite rhoptry kinase ROP16 that phosphorylates STAT3, leading to its translocation to nucleus and pro-growth response [42]. The more pronounced upregulation of *Socs1* relative to *Socs3* in our dataset implies that M2 macrophage polarization due to *T. gondii* infection is favoured [43]. While this study does not assess the effect of infection by a Type II strain, due to its expression of an inactive ROP16 kinase, manipulation of the macrophage immune defences likely occurs via alternative pathways and to a lesser extent [24]. Therefore, suppressors of cytokine signaling are not expected to be upregulated as markedly in an equivalent time course employing a Type II strain. Lastly, as was reported previously [23], upregulation of *Cd83* that is associated with antigen presentation took place regardless of anti-inflammatory modulation by Live *T. gondii*. This hints that *T. gondii* may allow some level of adaptive immune activation. However, given the parasite’s ability to suppress co-stimulatory signals and pro-inflammatory cytokines, this may also reflect a strategy of promoting a mature antigen-presenting phenotype that permits antigen display without eliciting a robust effector T cell response.

Additionally, our data revealed upregulation of other genes linked to proliferation. Notably, *Hbegf* is known to bind epidermal growth factor receptors (EGFR) to promote cell cycle progression and survival [44] and *Vegfa* facilitates macrophage migration [45]. Recently, *Nr4a3* was also labelled as an important mediator of increased migration through induction by ROP28 [36]. Taken together, these responses may help create conditions conducive to parasite survival, while simultaneously protecting the host cell from early cell death, potentially aiding parasite dissemination. Nevertheless, genes related to anti-infection processes such as autophagy regulation (*Plekhf1*) or neutrophil recruitment (*Cxcl2*) [46] were also induced. Despite these defenses, infection ultimately becomes established, indicating that the macrophage’s early response is not sufficient to halt *T. gondii.* In our dataset, these effects appear specific to live *T. gondii* infection, suggesting that the parasite triggers them through secreted factors and intracellular mechanisms. Other rapidly acting layers of regulation – such as translational control [47] – may also contribute, but remain to be explored.

Meanwhile, the transcriptional response of the invading *T. gondii* tachyzoite itself appears to be relatively modest and reflects a gradual shift in increased growth-related capabilities. We did not observe sudden decrease in *T. gondii* transcripts, nor surge of transcription, presumably because many secreted effector proteins required for immune evasion are already synthesized prior to invasion. Thus, the parasite effectively “pre-arms” for infection, and the outcome may be largely predetermined by these secreted factors prior host-parasite interaction. In favour of this hypothesis, rhoptry-related genes (ROP and RON) are upregulated more slowly, as evidenced by our 24-hour time point. Accumulation of these protein products awaiting post-translational processing at this time point has been reported previously [48]. A study by Claywell et al. proposes a mechanism of sensing egress timing [49], yet, it is not established whether these pathways interconnect and would therefore allow *T. gondii* to accurately time the upregulation of ROP and RON gene expression in preparation to the next round of infection. Overall, the parasite appears to rely on a steady, business-as-usual approach, rather than undergo a rapid, finely orchestrated, and environment-tailored shift of transcriptional program.

Several avenues warrant further investigation and harnessing single-cell transcriptomics represents a promising way to address the current limitations in our approach, particularly in light of variable parasite infectivity. Although our design maximizes the fraction of invaded macrophages, it also increases the number of *T. gondii* tachyzoites that fail to infect, potentially diluting the parasite-specific transcriptomic signal. Future efforts specifically dedicated to the parasite’s response might therefore consider lower parasite-to-host ratios (bulk analyses) or omit uninfecting parasite cells (single-cell resolution). Additionally, further work is needed to pinpoint specific parasite effectors that drive the observed transcriptional changes in macrophages, and applying proteomic techniques or knockout mutants might be most fruitful. Finally, while *in vitro* assays offer targeted mechanistic insights, they inevitably overlook the complexity of *in vivo* infections that involve multiple cell types and genetic variability in both host and parasite.

By comparing live *T. gondii* infection with dead-parasite stimulation, our study provides a unique perspective on the host response timing. We show that macrophages mount a pronounced immune reaction by the first hour, but this response is dampened specifically in the presence of live parasites, facilitating *T. gondii* establishment within the macrophage. This insight underscores the importance of early host-parasite interactions in shaping the outcome of infection and suggests new directions for dissecting these events.

## Methods

### Cell cultures

GFP-tagged *Toxoplasma gondii* type I strain RH (acquired from Frickel Lab) was used for all infection experiments. Human foreskin fibroblast (HFF) cells (ATCC) were used for routine *T. gondii* maintenance and expansion for infections. Mouse macrophage-like cell line RAW264.7 (ATCC) was passaged by scraping off, resuspension and 10-fold dilution when the culture reached approximately 80% confluence, which was typically every 3 days. HFFs were grown to near 100% confluence and passaged after trypsinization and 5- to 10-fold dilution. 100% confluence after passaging is achieved in approximately 1 week. Fully confluent HFFs were maintained for up to 2 weeks for further use. *Toxoplasma gondii* was maintained in HFFs at 90–100% confluence by passaging 10–100 µL (based on approximate visual estimation of parasite counts) of media containing extracellular tachyzoites from an infected T25 flask to an uninfected T25 flask. This procedure was carried out every 2–4 days. All uninfected and infected mammalian cell cultures were grown in Dulbecco’s Modified Eagle’s Medium – High Glucose (DMEM-HG) (Sigma) with addition of 10% initial volume of Fetal Bovine Serum (FBS; Gibco™) and 1% initial volume of 200 mM l-glutamine (Gibco™) in a 37°C incubator with a 5% CO_2_ atmosphere and 100% relative humidity. All mammalian cultures were routinely restarted from new cryostocks after they have reached passage number 15. All procedures involving work with mammalian cell lines and *T. gondii* were carried out in a BSL2 facility. iBMDM cells were prepared through Cre-J2 retroviral transduction [50].

### *T. gondii* preparation for infection time course

Before an infection experiment, *T. gondii* tachyzoite cultures in HFFs were expanded sequentially. First, when approximately 80% of HFFs have been lysed by the parasites in the T25 maintenance flask, a small aliquot was taken for further passaging while all the remaining extracellular parasites were used to infect a T75 flask of confluent HFFs. After 80% of HFF cells have lysed (typically between 24 and 48 hours post-infection), the culture supernatant was distributed between three T175 flasks with confluent HFFs. Finally, after another 24–48 hours, approximately 20% of HFF monolayer was lysed, at which point intracellular *T. gondii* tachyzoites were harvested for infection. The culture supernatant is removed and the tachyzoite-laden HFF monolayer was scraped off, resuspended in 5 mL of supplemented DMEM (pre-warmed to 37°C) for each T175 flask and transferred to a 15 mL polypropylene tube. The suspension was then passed through a 27G needle 4–6 times using gentle pressure on the plunger to break tachyzoites free. The parasites were then separated from debris by differential centrifugation: the suspension was centrifuged at 100 g for 5 min at room temperature, the supernatant was transferred to another tube followed by another centrifugation at 1000 g for 5 min at room temperature. The supernatant was carefully removed and the pellet containing the parasites was gently resuspended in 500 µL of pre-warmed medium. Parasite concentration was quantified by hemocytometry of a 100-fold dilution in phosphate-buffered saline (Gibco). One T175 HFF flask routinely yielded 2 × 10^8^ to 4 × 10^8^ purified intracellular tachyzoites. Dead *Toxoplasma* for control infection was prepared by performing five freeze-thaw cycles in dry ice–ethanol slurry and warm water. Full lysis of the parasites was confirmed by observation under a microscope.

### Macrophage infection

At approximately 80% confluence, RAW264.7 or iBMDM cells were mechanically scraped off the bottom of the flask and resuspended in 10 mL of pre-warmed medium and quantified using hemocytometry. A T75 flask routinely yielded about 4 × 10^7^ cells. Cells were diluted to the desired concentration with medium and seeded into multi-well tissue culture plates. 2.5 mL or 0.5 mL of medium was used for standard 6-well plates and 24-well plates, with each well containing approximately 500K or 100K cells, respectively. After seeding, the cells were allowed to adhere to the bottom of the plate for six hours to overnight before infection. One plate per time point was prepared. For infection, the medium was removed, and the parasite suspension (1.2 × 10^7^ tachyzoites in 200 µL of medium per well in a 24-well plate) was immediately added to the wells. For Mock or Dead *Toxoplasma* infections, 200 µL of medium or lysed *T. gondii,* respectively, were used. Then, the plates were stacked and gently rocked by hand in a forward-backward and then left-right motion for 30 s to distribute the added tachyzoites. The plates were placed in the incubator unstacked. After 15 min, the plates were removed from the incubator. This was considered time point zero for the infection [51]. The parasite suspension was removed from the wells followed by two washes (500 µL of pre-warmed medium each) to remove unattached tachyzoites. Another 500 µL of medium was added and further incubation was carried out in the incubator until the desired time point, when the medium was removed and a single wash with 500 µL of PBS was performed. After the removal of PBS, the wells were promptly imaged with ZOE Fluorescent Cell Imager (BioRad) in brightfield and green channels and the cells were immediately lysed by addition of 200 µL of TRIzol™ Reagent (Invitrogen™). The lysates were then stored at −20°C. Total RNA was extracted according to manufacturer’s guidelines, dissolved in 10 mM Tris-HCl (pH 7.5) and quantified using NanoDrop™ One. RNA integrity was confirmed on 2% agarose TBE gels. The RNA was stored at −80°C until further use.

### Infection efficiency quantification

To quantify the fraction of macrophages that were infected, micrographs from brightfield and green channels were merged with ImageJ and cells were manually counted using an ImageJ plugin Cell Counter [52]. Infection efficiency was defined as the following:


$$E_{inf}=\frac{n_{infected}}{n_{all}}\times \text{1}\text{0}\text{0}\%$$ (1)

Where E_inf_ is the infection efficiency in percent, n_infected_ is the number of macrophages in a micrograph viewing field that colocalize with GFP fluorescence of the parasites, n_all_ is the number of all the macrophages present in that field. Micrographs were taken in the centre of each well. Three random non-overlapping fields were quantified, and the average infection efficiency value was calculated.

### qRT-PCR

Quantitative reverse transcription PCR was used to verify expected infection responses and validate RNA sequencing results. Total RNA was reverse-transcribed with M-MLV reverse transcription (RT) enzyme (Clontech), MMLV RT 5X buffer (Promega), and random hexamers (Promega) in the presence of RNaseOUT™ (Invitrogen™) ribonuclease inhibitor. The synthesized cDNA was diluted five times. 2 µL of cDNA per well were used in 384-well plates (Applied Biosystems), along with iTaq™ Universal SYBR® Green Supermix (BioRad) and 0.5 µM final concentrations of forward and reverse primer (for primer sequences see S1 Table). Primers against *T. gondii* genes were designed using NCBI Primer–BLAST to span exon-exon junctions. Reference gene and target gene reactions for three biological replicates per condition were performed in Applied Biosystems ViiA™ 7 Real-Time PCR System on fast mode. Analysis was performed using the ΔΔCt method [53] comparing 15 min and 120 min timepoints. Assays were controlled for species specificity, genomic DNA amplification and ambient contamination.

### PARFA-Seq library preparation

PARFA-seq is an open-source library preparation protocol that is time-efficient, features dual indexing for multiplexing many samples and was tested to perform well with reagents commonly available in molecular biology laboratories (S3 Fig in S1 File). It involves reverse transcription of poly-A(+) RNAs, second strand synthesis by random priming and two rounds of PCR to attach necessary handles. Library quality control is also simple to assess through standard non-denaturing gel electrophoresis (Fig S4 in S1 File). As *T. gondii* transcriptome is also polyadenylated, both the host and the parasite transcriptomes were simultaneously captured. Upon demultiplexing and adapter trimming, reads are mapped to a combined *T. gondii*–mouse genome and unique mappers are used for count matrix generation.

Libraries for transcript quantification by PARFA-Seq were prepared using total RNA from the RAW264.7 infection time course. The primer 3PS-RTP was annealed to poly-A(+) RNA, which was then reverse transcribed with SuperScript™ III (Invitrogen™). Samples were then treated with RNase H (Thermo Scientific™). Unincorporated oligonucleotides were removed using Sera-Mag™ magnetic beads (Sigma) according to manufacturer’s protocol. Oligonucleotide PARFA-uRPI-# containing a random hexanucleotide at the 3′ end was annealed to the cDNA, allowing the synthesis of the second DNA strand using NEBNext® Ultra™ II Q5® Master Mix (NEB) followed by addition of RPI-# and PCR for five cycles. Each library was prepared with a unique combination of PARFA-uRPI-# and RPI-#. PCR products were then size-selected using Sera-Mag™ magnetic beads at 0.65 and 1.00 ratio of beads to sample by volume. DNA after size selection was then amplified using primers RP1 and RPI-# using the same master mix for another 12–15 cycles, followed by second round of size selection using the same parameters. Library quality was verified by TBE-PAGE in 0.75 mm 6% gels. Gel images were used to estimate DNA concentration based on band intensity in the region of interest (approximately 350–500 bp). Image Studio™ Lite software (LI-COR Biosciences) was used for this purpose. Equal amounts of each library were pooled (1 pool per replicate). Library pools were further size-selected by gel electrophoresis: 1 mm precast 10% TBE-PAGE gel (Invitrogen™) were loaded with pooled libraries, run for 40 min at 200 V. The gel was stained with SYBR™ Gold (Invitrogen™) for 5 min, visualized in blue LED light and 350–500 bp regions were excised and subjected to DNA extraction. Briefly, slices containing DNA of interest were crushed, extracted with 500 μL of DNA extraction solution (300 mM NaCl, 10 mM Tris pH 8.5, 1 mM EDTA) overnight. Next day, the supernatant was filtered off and mixed with 2 μL of GlycoBlue™ coprecipitant (Invitrogen™), 500 μL of isopropanol, and allowed to precipitate at −20°C for 1 h to overnight. The DNA was pelleted by centrifugation (4°C, > 20 000 g for 1 h) and washed twice with cold 80% ethanol. The precipitate was then air-dried and dissolved in 10 μL of 10 mM Tris (pH 8.5, nuclease-free). The libraries were quantified with Qubit™ 4 fluorometer and dsDNA HS Assay Kit (Invitrogen™). Replicates 2 and 3 were pooled together in equimolar ratio and mixed with additional libraries to increase complexity. All library preparation procedures were performed in 0.5–2.0 mL DNA LoBind® tubes (Eppendorf), where applicable. A scheme of PARFA-Seq library preparation is given in Fig 2A.

### Next-generation sequencing and read pre-processing

Libraries were sequenced in Illumina® NextSeq® 500 instrument (1 × 75 bp mode) by Cambridge Genomic Services. The reads were output from the Illumina® sequencing platform and grouped by the 3′ barcode. The resulting data were then sorted based on a 5′ barcode, which was then trimmed from the reads. All 5′ barcode trimming and UMI manipulation was done using *umi_tools* [54]. The remaining poly-A sequences (5 nt or longer) were removed with *bbduk* [55]. Filtered reads were aligned to the m10 genome of *Mus musculus*, combined with *Toxoplasma gondii* genome built with ME49 release 29 using *STAR* aligner [56]. PCR bias was accounted for using *umi_tools dedup*: if multiple reads with the same UMI are aligned in the same position, only one read is retained. To generate a read count table, each sample was passed through *htseq-count* [57] and reads that aligned to exactly one feature in the GTF file were counted.

### Downstream transcriptome analysis

Genes with fewer than ten counts over all samples were filtered out. *DES*eq2 package [58] was used for differential expression analysis. To deconvolute expression data (in particular, Log_2_(Fold change)), non-negative Matrix Factorization (NMF) was selected because of intuitive interpretation as well as its ability to assign genes to every component quantitatively by their weight scores, as opposed to qualitative clustering (e.g., k-means, where each gene belongs to one cluster only). NMF R package [59] was used to cluster genes of similar dynamics into components and extract the dominant features in each component. Mouse and *T. gondii* gene sets of interest were screened for GO-term enrichment using *gprofiler2* [60] and *ToxoDB* [61], respectively.

## Supporting information

S1 File Supplementary Figures. Figures S1 to S8.
(PDF)

S1 Table Oligonucleotides used in this study. In RPI-# and PARFA-uRPI‑#, nucleotide stretches marked by X represent barcode fragments. Accordingly, # indicate all the corresponding primers used that only differ by barcode sequences.
(XLSX)

S2 Table Summary of all significant gene enrichment terms for every condition (as combination of sample type and timepoint). (XLSX)
