# Supplementary material for: Early upregulation of immune suppressors dominates the macrophage response to Toxoplasma gondii
Source: PLoS One. 2025 Nov 24;20(11):e0336849. doi: 10.1371/journal.pone.0336849 (PMC12643308; doi:10.1371/journal.pone.0336849)
Supplement: S1 File — Figures S1 to S8. (PDF) [file pone.0336849.s001.pdf]

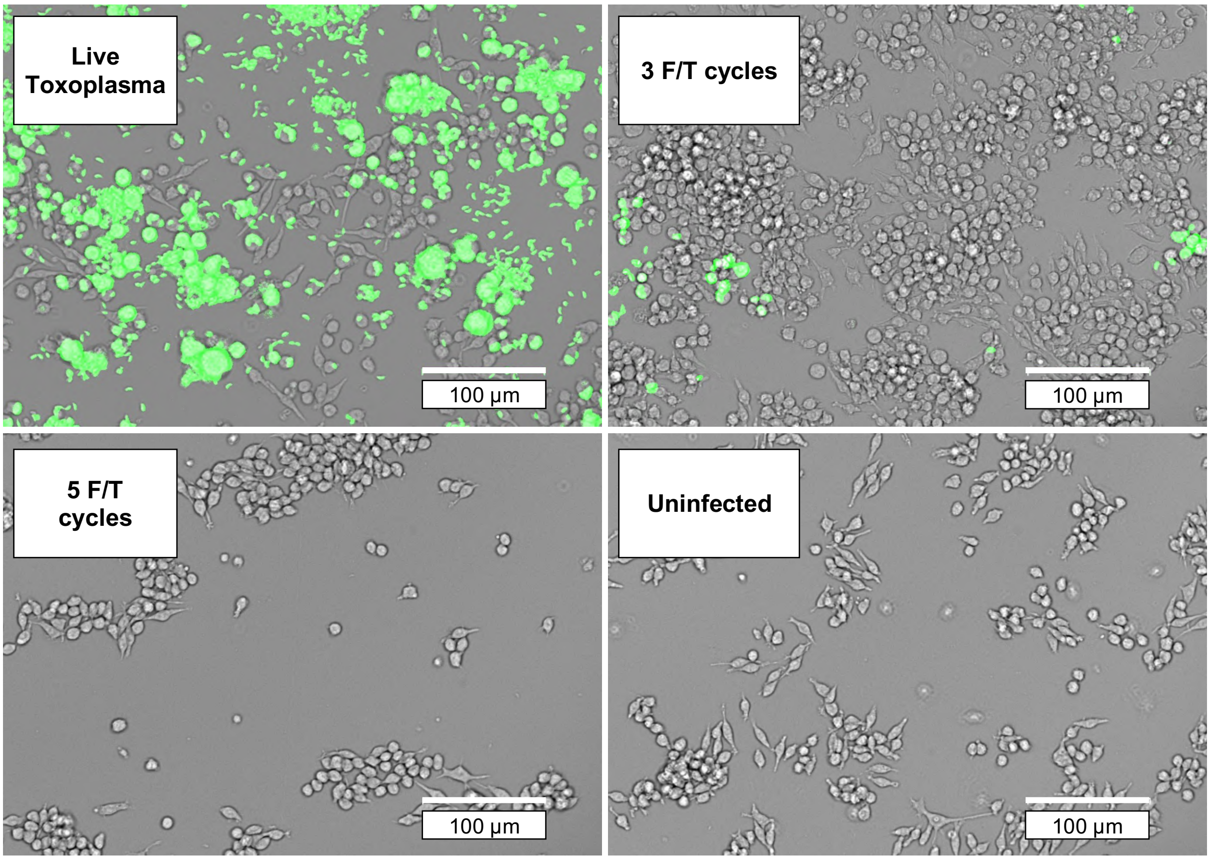

Figure S1. Validation of *Toxoplasma gondii* inactivation by freeze-thawing. Green fluorescence overlay indicates *T. gondii* grown overnight. 5 F/T cycles were sufficient to fully inactivate the tachyzoite preparations.

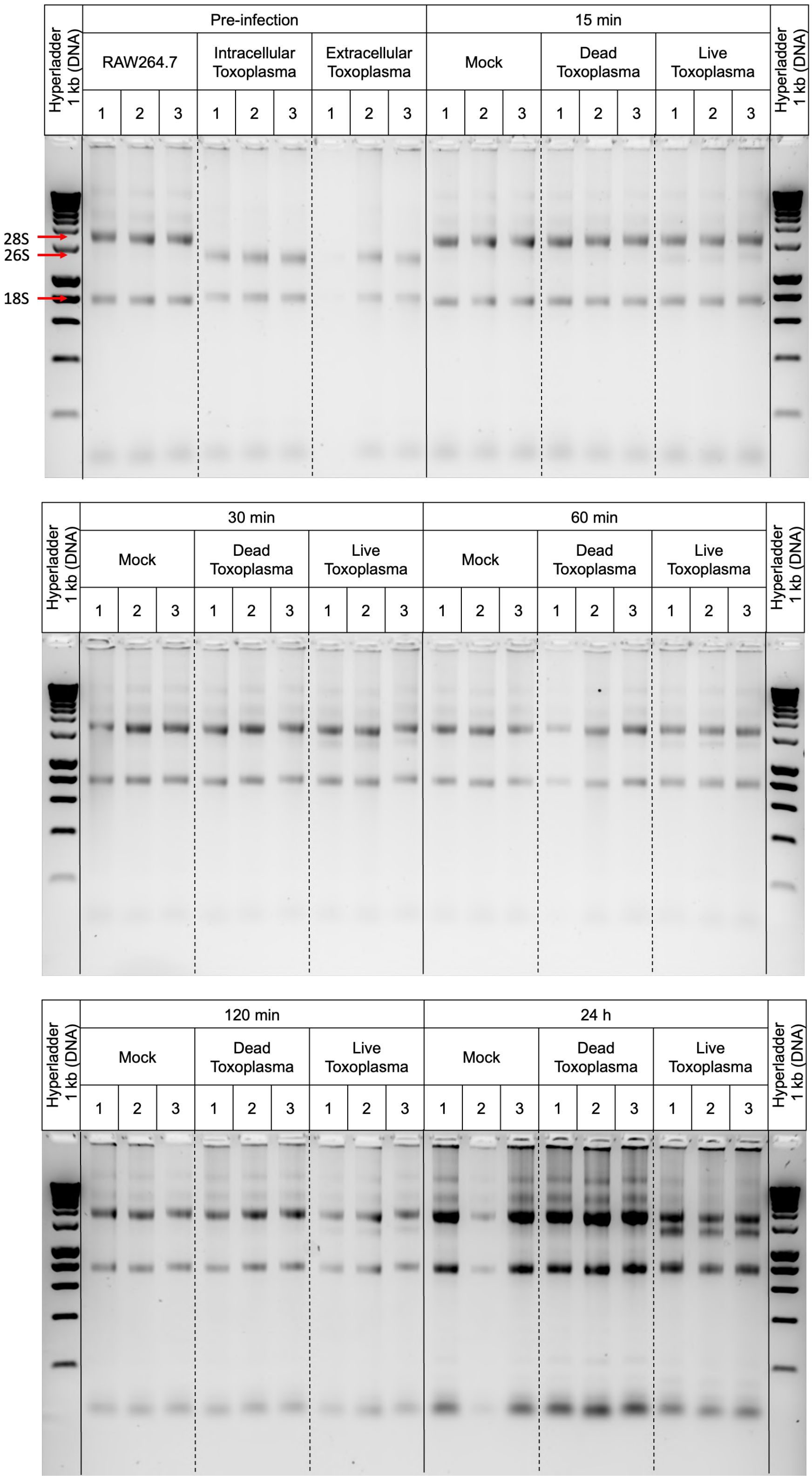

Figure S2. Quality control of extracted RNA from time course samples on 2% agarose gels. Expected 28S (mouse), 26S (*T. gondii*) and 18S rRNA band positions are marked with red arrows. By 24 h, 26S rRNA relative amount has increased substantially indicating parasite expansion. Intact rRNA bands and absence of a smear caused by short RNAs indicated sufficient RNA quality.

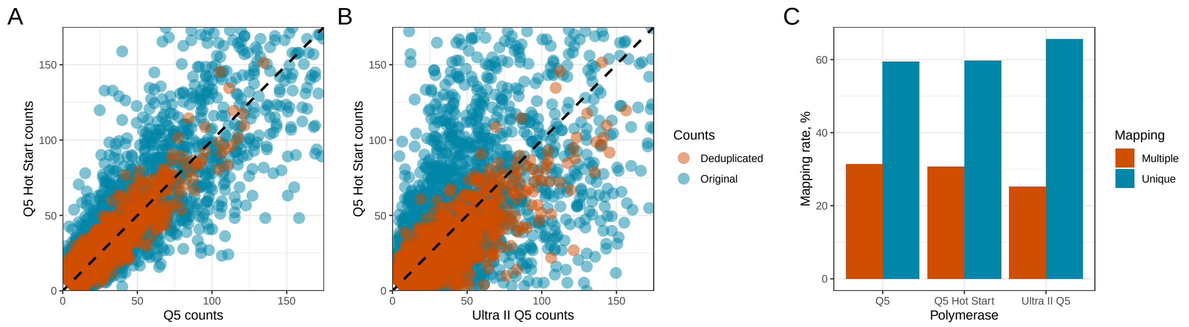

Figure S3. Comparison of PARFA-seq library preparation using different PCR reagents. **A** and **B**: Raw and deduplicated count distribution for Q5-Q5 Hot Start and Ultra II Q5-Q5 Hot Start pairs, respectively. **C**: mapping statistics for each library.

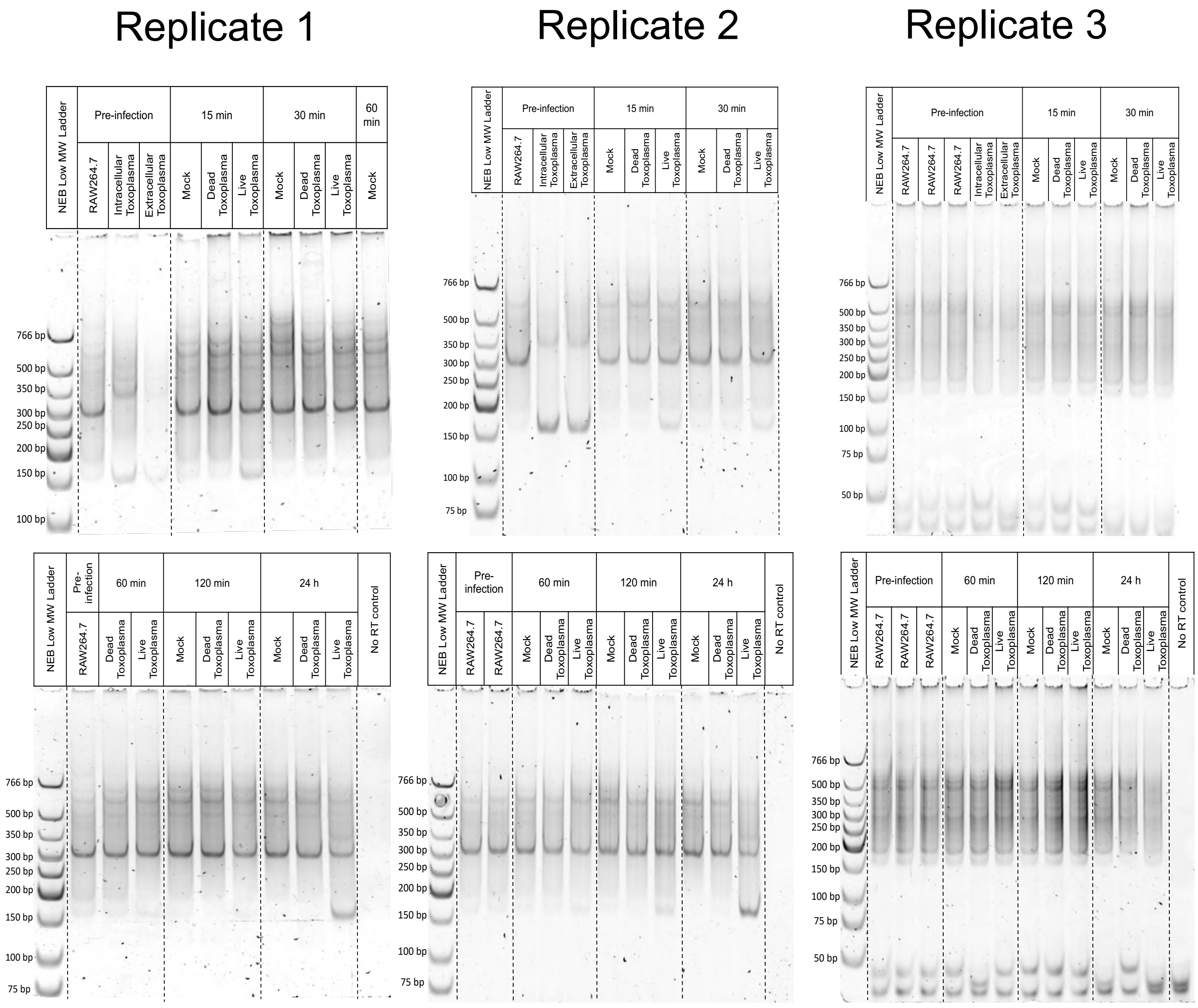

Figure S4. PAGE quality control of time course PARFA-seq libraries in preparation before pooling and final size selection.

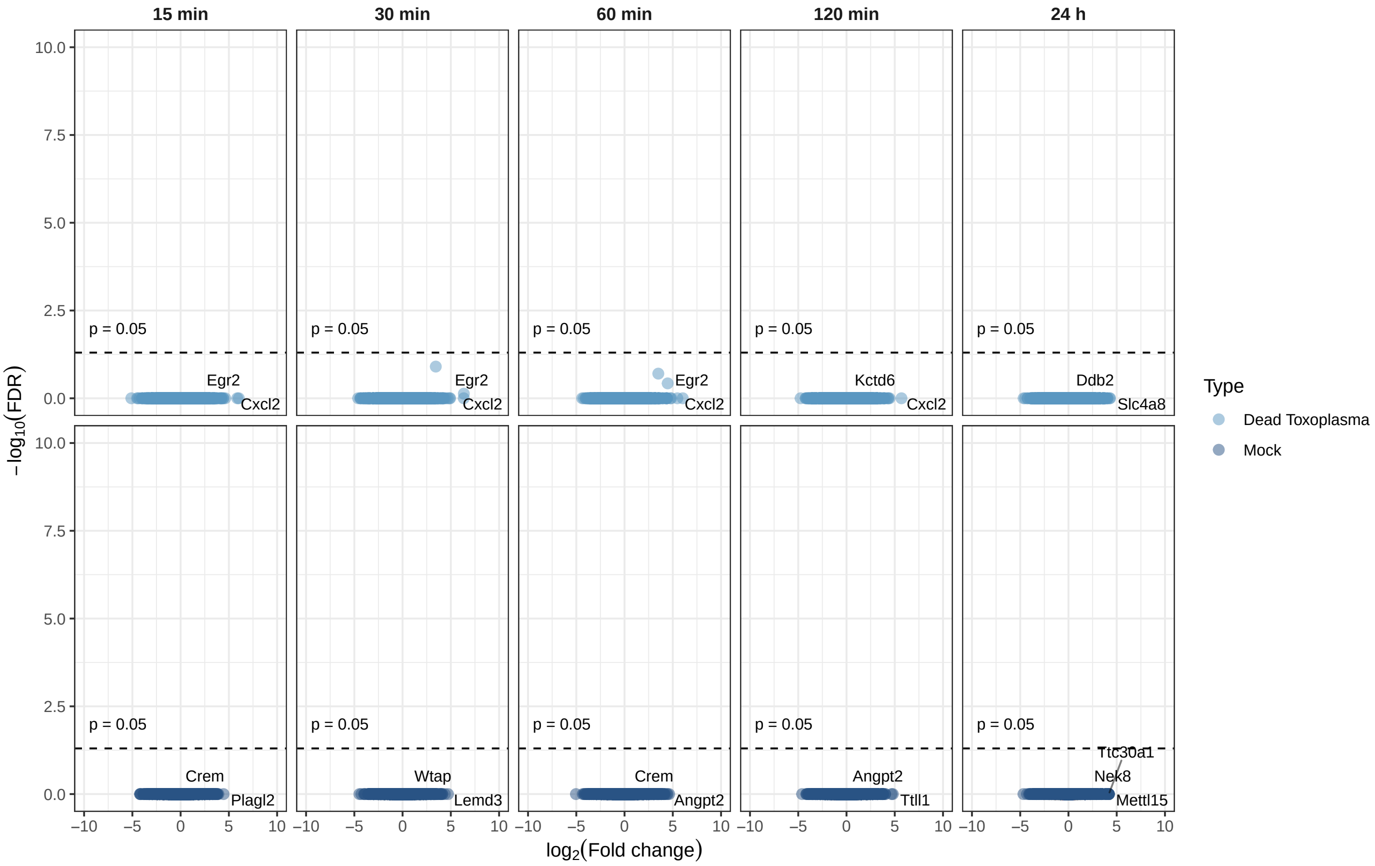

Figure S5. Differential expression of Dead Toxoplasma and Mock samples throughout the time course. Dashed line indicates p-value threshold of 0.05. Top upregulated genes are labelled.

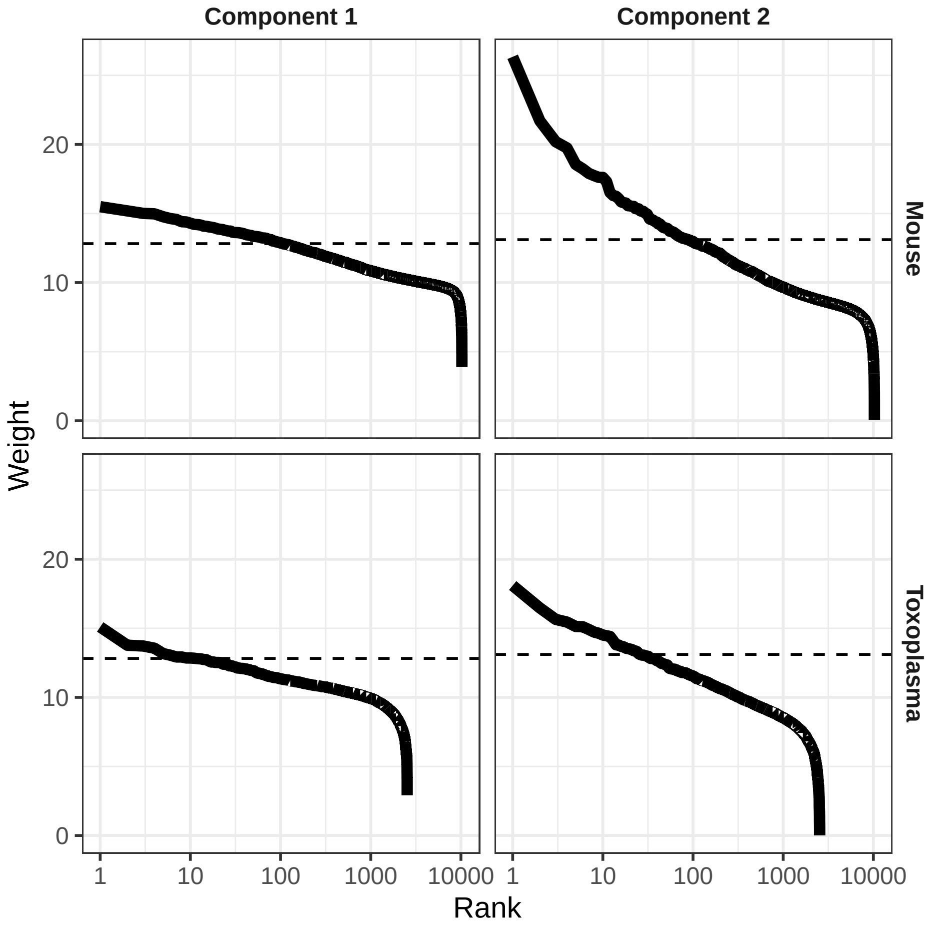

Figure S6. Mouse and *T. gondii* genes ranked by weight for each component from non-negative matrix factorization (mouse + *T. gondii* co-transcriptome, 15 min-120 min). Dashed line indicates weight threshold to subset top features for gene set enrichment analysis.

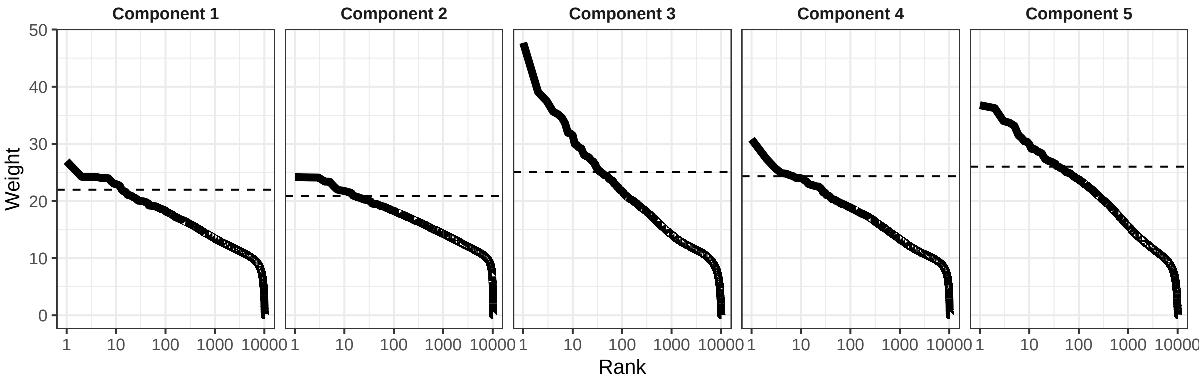

Figure S7. Mouse genes ranked by weight for each component from non-negative matrix factorization (mouse transcriptome only, 15 min-24 h). Dashed line indicates weight threshold to subset top features for gene set enrichment analysis.

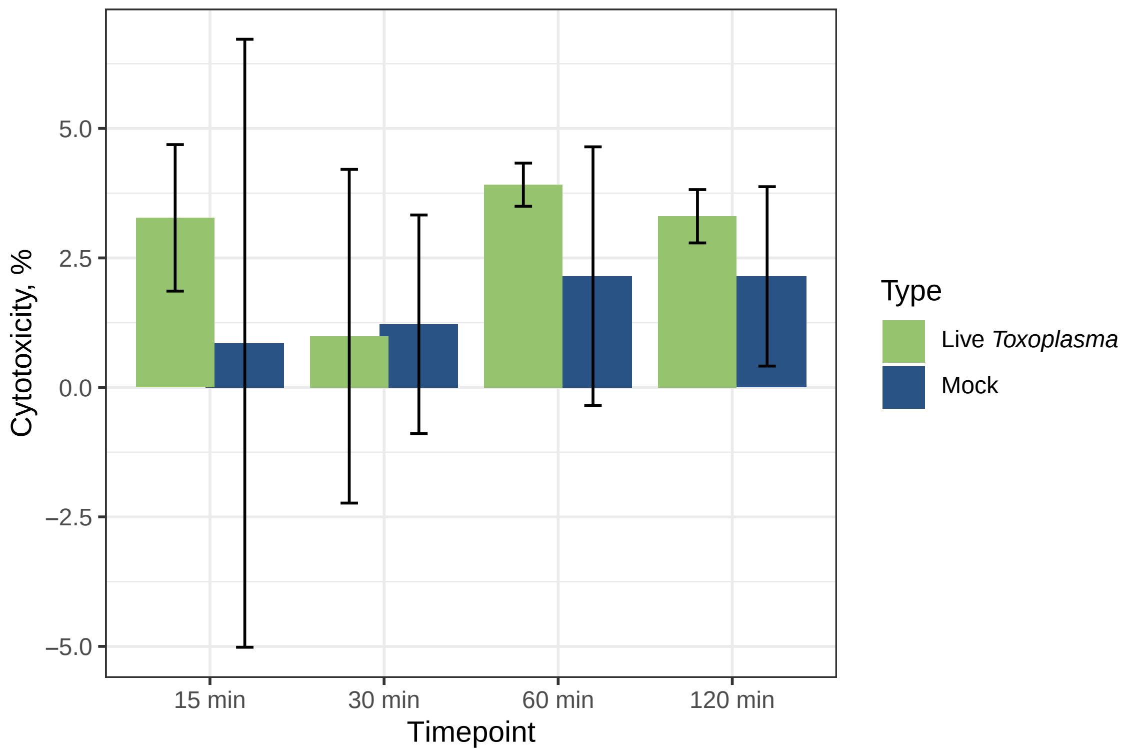

Figure S8. Cytotoxicity values upon Mock and Live *Toxoplasma* infection, determined based on lactate dehydrogenase release into media. Error bars indicate standard error.
